# Closure Of Thoracic Wall Defect Using Breast Implant Capsule Tissue As A Rotation Flap - A Case Report

**DOI:** 10.1080/23320885.2018.1476150

**Published:** 2018-05-24

**Authors:** Michael Josiassen, Madeline T. Kudibal, Christina Gramkow, Stig-Frederik Trojahn Kølle

**Affiliations:** Department of Plastic Surgery and Burn treatment, Rigshospitalet, Copenhagen, Denmark

**Keywords:** Thoracic flap, iatrogen pneumothorax

## Abstract

A 70-year old woman underwent thoracic surgery resulting in rupture of a silicone implant. During re-implantation at a later time an iatrogenic thoracic wall defect was discovered. In order to complete re-implantation the defect was closed using a rotation flap constructed from capsular tissue from the previous silicone implant.

## Introduction

Iatrogenic injury to the thoracic wall and pneumothorax is a common side effect to thoracic or pulmonary procedures, yet seldom treated surgically whilst performing open surgery [1]. Defects of the thoracic wall and parental pleura are commonly managed by soft tissue coverage by primary closure, local flaps or insertion of a synthetic mesh in cases where additional stability is required [2]. In this paper, we report a case of an initially undiagnosed iatrogenic thoracic wall injury during thoracic surgery, which was later discovered while performing unilateral breast augmentation. We further present a not previously described technique in closing a thoracic wall and pleural defect, using a rotation flap created from capsular tissue from a breast implant.

## Case report

A 70-year-old female was referred to breast re-implantation of the right side due to iatrogenic rupture and explantation during thoracic surgery. The patient had a medical history of cancer in the right breast in 1984 with subsequent treatment including unilateral mastectomy, radiochemotherapy and secondary reconstruction with a subpectoral silicone implant. In 2015 the patient was diagnosed with leiomyosarcoma in the right thigh and bilateral pulmonary metastases. The patient underwent video-assisted thoracoscopic surgery (VATS) wedge resection of the right lung and during this procedure iatrogenic perforation of the silicone implant and injury to the thoracic wall occurred. The thoracic cavity was closed and the perforated breast implant removed without diagnosis of the defect.

In stabile condition, the patient expressed a strong desire for breast re-implantation and during this procedure the defect of the thoracic wall and parietal pleura was observed. As the defect was minor, simple closure of soft tissue over the defect would be appropriate in patients not planned for breast reconstruction, however the patient in this case had expressed an extremely strong desire for breast reconstruction. In case of direct closure communication would persist between the lung and the capsular space and therefore it was necessary to close the defect with a thin layer of material allowing both secure closure of the defect and re-implantation. The highly vascularised capsule is known to be appropriate for creation of flaps and grafts, and has previously been used in management of issues in relation to breast implants [3]. The capsule tissue was an appropriate choice for creation of a pedicled flap in this case because the tissue presented a minimally invasive treatment option for the patient compared to closure with a muscle flap or addition of non-autologus tissue such ADM, increasing infection risk. The anterior part of the capsule in relation to the pectoral muscle was dissected free from the upper pole to the inframammary fold resulting in a medially and centrally vascularised flap. The flap was rotated with the agglutinating side facing downwards to cover the pleural defect and sutured shut. The flap was not expected to be airtight, however placement of the implant directly over the flap ensured necessary pressure to prevent displacement and air leaks. Pressure ventilation found the new capsular patch airtight. Next pleuracentesis was performed in order to control the pneumothorax and finally the flap was sutured in place to close the defect before placement of the silicone implant. Preventative iv. antibiotics were administered. Postoperatively the patient had discrete air effusion once the chest tube suction was discontinued and after five days chest x-ray showed a 5,2 cm apical lung pneumothorax. The patient had no pulmonary symptoms. At the one-week follow-up a regression of the pneumothorax from 5,2 cm to 3,1 cm was found and at the twelve month follow up the reconstruction was fully functioning and the patient asymptomatic.

## Discussion

The standard treatment of pneumothorax is highly dependent on the patient’s clinical condition, size of pneumothorax and underlying disease [4]. To use the highly vascularised breast implant capsule tissue for reconstructive purposes was first described in the early 1993 by Bengtson [5] and its use has since been described as appropriate as both as a flap and as a graft [3]. The indication for using the capsule tissue is mainly to address breast implant related issues, however permanent lip augmentation and a myocapsular flap for pharyngeal reconstruction has also been described [3,6,7]. Advantages of creating local flaps from capsular tissue include sparing functional tissue such as muscles and to avoid placement of non-biological material. The thin but rigid structure of the capsule can be used for addressing a number of issues related to breast implants including repositioning and coverage. When considering using the capsule for flaps or grafts it is important to consider vascularity and thickness. Both are dependent on the time from primary breast implant placement and vascularity has been found to be higher in contracted capsules than non-contracted capsules. A disadvantage of using the capsular tissue is that the persistence is unknown, as available studies have reported varying results [3]. In this case we performed a novel treatment method due to the unique perioperative problem but also due to the patient’s strong desire for re-implantation. The anterior capsule rotation flap proved tight, re-implantation was successful and the patient suffered no postoperative complications. We thus report a potential solution to closing a pleural defect with an anterior capsule rotation flap; however, similar examples are yet to be described.
